# Comparison of Three Chemotherapy Regimens in Elderly Patients with Diffuse Large B Cell Lymphoma: Experience at a Single National Reference Center in Mexico

**DOI:** 10.1155/2016/9817606

**Published:** 2016-07-10

**Authors:** Diana Nolasco-Medina, Nancy Reynoso-Noveron, Alejandro Mohar-Betancourt, Alejandro Aviles-Salas, Osvaldo García-Perez, Myrna Candelaria

**Affiliations:** ^1^Hematology Department, Instituto Nacional de Cancerología, 14080 Mexico City, DF, Mexico; ^2^Clinical Research Division, Instituto Nacional de Cancerología, 14080 Mexico City, DF, Mexico; ^3^Instituto de Investigaciones Biomédicas, UNAM, 04510 Mexico City, DF, Mexico; ^4^Pathology Department, Instituto Nacional de Cancerología, 14080 Mexico City, DF, Mexico; ^5^Nuclear Medicine Department, Instituto Nacional de Cancerología, 14080 Mexico City, DF, Mexico

## Abstract

*Background.* Although chemotherapy added to rituximab is a standard of care for diffuse large B cell lymphoma (DLBCL), treatment of patients ≥65 years of age remains controversial due to comorbidities.* Methods*. This is a retrospective, comparative, nonrandomized study of patients ≥65 years of age, who were diagnosed with DLBCL but not previously treated. Demographic characteristics and comorbidities were analyzed. Three rituximab-containing treatment regimens (standard RCHOP, anthracycline dose-reduced RChOP, and RCOP) were compared. Descriptive analyses were conducted. Survival was calculated with the Kaplan-Meier method, and differences were compared with the log-rank test.* Results*. In total, 141 patients with a median age of 73.9 years were studied. The three treatment groups had comparable demographic characteristics. The overall response was 77%, 72.5%, and 59% in groups treated with RCHOP, RChOP, and RCOP, respectively. After multivariate analysis, the factors influencing the overall survival were the presence of B symptoms, poor performance status (ECOG ≥ 3), and febrile neutropenia. Factors influencing disease-free survival were febrile neutropenia, high-intermediate and high-risk IPI scores, and treatment without anthracycline.* Conclusion*. A higher ORR (overall response rate) was achieved with standard RCHOP, which influenced DFS and OS, although it was not statistically significant compared with the other groups. Interventional phase 3 trials testing new molecules in patients aged 70 to 80 years and older are required to improve the prognosis within this growing population.

## 1. Introduction

The prevalence of non-Hodgkin lymphoma (NHL) has increased in recent years, and it is now estimated to be the 12th leading cause of death among patients with cancer in the United States [1]. Diffuse large B cell lymphoma (DLBCL) is the most common lymphoid malignancy worldwide, with approximately 40% of cases occurring in patients over 70 years of age [2–5]. The actual standard of care includes the anti-CD 20 monoclonal antibody rituximab in combination with chemotherapy [6–8], in all age groups, including elderly patients. As shown in the randomized phase III trial by the* Groupe d'Etudes des Lymphomes de l'Adulte (GELA)* in patients aged 60–80 years, the combination of CHOP and rituximab was significantly superior to CHOP alone in terms of complete response rate and survival, without a clinically significant increase in toxicity [8]. However, in elderly patients, the presence of comorbid diseases, impaired bone marrow function, and altered drug metabolism may increase the number of treatment-related complications, as well as hospitalization and mortality rates. Frailty is a well-defined syndrome, a clinically recognizable state with increased vulnerability resulting from aging-associated declines in reserve and function across multiple physiologic systems [9]. To date, there has been no standardized consensus regarding the management of elderly patients with DLBCL, especially for the frail population. In fact, in its latest version, the NCCN proposed regimens with attenuated or complete elimination of anthracyclines as a first-line treatment for frail patients and those with poor left ventricular function [10, 11]. A lack of evidence of a standard treatment modality that should be considered in elderly patients in the presence of comorbidities was the motivation to conduct this retrospective study; the goal of the study was to determine whether standard RCHOP, reduced anthracycline RChOP, or RCOP could be preferred regimens for patients older than 65 years in terms of response, toxicity, and impact on overall survival.

## 2. Material and Methods

A retrospective, comparative, nonrandomized study was conducted to evaluate three treatments in terms of response, toxicity, relapse rate, and survival. The main objective of this study was to determine which of these treatments could be recommended for elderly patients, first in terms of overall survival (OS) and second in terms of toxicity and response rate.

The inclusion criteria were patients ≥65 years of age with a histologic diagnosis of DLBCL, who were treated at a single national reference institution (Instituto Nacional de Cancerología, Mexico) from January 2011 through January 2015. The Ethics Committee approved the review of clinical files.

Clinical characteristics included age, gender, comorbidities (diabetes mellitus, blood arterial hypertension, cardiopathy, history of hepatitis, and HIV status), and the presence of B symptoms.

Histological classification was performed by a Hans nomogram and was based on the expression of CD10, BCL6, and MUM1 as previously described [10]. Briefly, samples expressing CD10 (+) or CD10 (−), BCL6 (+), and MUM1 (−) were defined as the germinal center (GC); those with CD10 (−), BCL6 (−) or CD10 (−), BCL6 (+), and MUM1 (+) were considered as nongerminal centers.

All three treatment regimens were administered every 21 days as follows.

*RCHOP.* Rituximab (MabThera®) 375 mg/m^2^, cyclophosphamide 750 mg/m^2^, doxorubicin 50 mg/m^2^, vincristine 1.4 mg/m^2^ (maximum dose 2 mg) and prednisone 100 mg/day/5 days.

*RChOP.* Rituximab (MabThera) 375 mg/m^2^, cyclophosphamide 750 mg/m^2^,* doxorubicin 25 mg/m* ^*2*^, vincristine 1.4 mg/m^2^ (maximum dose 2 mg), and prednisone 100 mg/day/5 days were administered.

*RCOP.* Rituximab (MabThera) 375 mg/m^2^, cyclophosphamide 750 mg/m^2^, vincristine 1.4 mg/m^2^ (maximum dose 2 mg), and prednisone 100 mg/day/5 days were administered.

Antiemetic therapy with 5HT3 antagonists was given with each cycle. Prophylactic granulocyte colony-stimulating factor (G-CSF) was not used routinely; in most cases, it was indicated for patients after developing grade III-IV neutropenia or febrile neutropenia.

The response to treatment was evaluated using standard international criteria. For patients in whom PET-CT was performed before and after treatment, the Deauville criteria were used [21]; in patients with increased glucose blood levels (>170 mg/dL) that contraindicated PET-CT, CT alone was performed, and the response was evaluated by the standard Cheson criteria [22]. Toxicity was assessed by the Common Terminology Criteria for Adverse Events, which is usually applied in hematooncology clinical trials (CTCAE, version 4.0) [19].

Descriptive analyses were performed for demographic and clinical characteristics. The significant differences between groups were assessed with the chi-square test;* k* independent samples were used for categorical variables, and the Kruskal-Wallis test was used for quantitative variables with free distribution.

Survival was calculated with the Kaplan-Meier method and differences between subgroups were compared with the log-rank test. Cox regression analysis was used to identify the effect of treatment regimens and was adjusted by clinical characteristics of relapse rate and overall survival. A value of *p* < 0.05 was considered statistically significant. All analyses were performed using STATA V.12.

## 3. Results

### 3.1. Demographics

A total of 141 patients, with a median age of 73.9 y (range 66–81 y), were included. As shown in Table 1, the patients were grouped by treatment regimen as follows: 53 (37.6%) patients received standard doses of RCHOP (Group A), 48 (34%) received a regimen with lower doses of an anthracycline (RChOP) (Group B), and 40 (28.3%) were treated without an anthracycline (RCOP) (Group C). When comparing demographic and clinical characteristics among the three groups, only a higher frequency (32.5%) of diabetes mellitus was documented in Group C (*p* = 0.042). No other significant difference was found among the 3 treatment regimens for the following variables: blood pressure, cardiopathy, left ventricle ejection fraction, hepatitis B or C, HIV status, the presence of B symptoms, clinical stages III-IV, and baseline performance status (evaluated by ECOG score). Most were considered as high-intermediate or high-risk (68%) according to the age-adjusted IPI scale.

Based on the histological classification, a higher proportion of the GC subtype was documented in RCHOP patients (54% versus 29.2% and 40% in RChOP and RCOP groups, resp.); this difference was statistically significant (see Table 1).

### 3.2. Response

Overall response rate (ORR = CR + PR) was achieved in 77% (*n* = 41/53), 68.7% (*n* = 33/48), and 60% (*n* = 24/40) in Groups A, B, and C, respectively. Although ORR was higher in Group A, this difference did not achieve statistical significance, which could be due to the sample size in each group.

### 3.3. Toxicity

As shown in Table 2, 52% (*n* = 28/53), 35% (*n* = 17/48), and 60% (*n* = 24/40) of patients treated with RCHOP, RChOP, and RCOP, respectively, did not experience severe toxicity. Myelosuppression was absent in approximately two-thirds of patients; only 20.75% (*n* = 11/53), 20.8% (*n* = 10/48), and 22.5% (*n* = 9/40) of patients experienced myelosuppression requiring transfusion in Groups A, B, and C, respectively. Overall, the frequency of severe toxicity was similar in the three groups, although febrile neutropenia was more frequent in patients receiving either whole or reduced doses of anthracycline (20.6% (*n* = 12/53) and 22.92% (*n* = 11/48) for RCHOP and RChOP, resp.), when compared with the group without an anthracycline (10%, *n* = 4/40); however, this was not statistically significant (*p* = 0.09).

### 3.4. Survival

**Figure 1:**
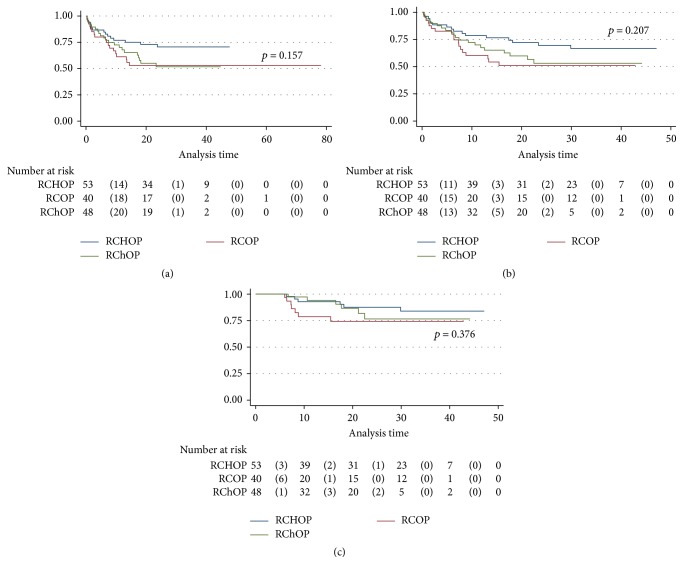
(a) Overall survival by treatment group. (b) Disease-free survival by treatment group. (c) Relapse-free survival by treatment group, considering only responder patients.

Median follow-up in the entire population was 19.9 months (7.16 and 33.26 months for the 25th and 75th percentiles, resp.). As shown in Figure 1, higher disease-free survival (DFS) and overall survival (OS) were observed in patients treated with RCHOP. In this group, the median DFS and OS were not achieved. However, a median DFS of 21 and 18 months and median OS of 22 and 19.5 months were documented in the RChOP and RCOP groups, respectively.

After analyzing only the subgroup of responder patients, DFS was similar among the three treatment groups; 75% of patients had no relapse at 17 months of follow-up (Figure 1(c)).

Cox regression analysis was performed to determine the factors influencing OS and DFS. After the univariate analysis, these factors were as follows: ECOG ≥ 2, B symptoms, bulky disease, high IPI score, anemia (hemoglobin < 8 g/dL), elevated LDH, involvement of ≥3 extranodal sites, increased creatinine (>2 mg/dL), hypoalbuminemia (<3 g/dL), elevated beta-2 microglobulin, presence of infectious complications, required hospitalization, febrile neutropenia, and incomplete cycles of treatment. However, after the multivariate analysis, only the following factors remained significant for OS: ECOG ≥ 3, involvement of ≥3 extranodal sites, presence of B symptoms at diagnosis, febrile neutropenia, and incomplete cycles of treatment (see Table 3). Similarly, febrile neutropenia, incomplete treatment, and high-intermediate and high risk IPI score had also a negative impact on DFS in the multivariate analysis (see Table 4). Regarding the impact of treatment on DFS, if we include only those patients who achieved a response, the small number of relapses resulted in a broad IC in the HR (as shown in Table 4). However, after including relapsed patients and nonresponders in the multivariate model of DFS, the HR of RCOP versus RCHOP of 0.966 (95% CI 0418–2234) was close to the estimated OS. The small sample in each group could be a reason why none of the differences were statistically significant.

## 4. Discussion

The study of cancer and aging is emerging as a critical issue in oncology. Patients with DLBCL vary in clinical presentation, prognosis, and response to current therapies. Treatment with RCHOP has been determined as the standard of care in prospective trials [8, 12] that included a minimal proportion of elderly patients; the inclusion of patients with comorbidities was also limited. Therefore, some authors have proposed attenuated immunochemotherapy regimens for such patients, either without an anthracycline [16], a reduced anthracycline [13, 16], or anthracycline and cyclophosphamide doses [14, 18], or including pegylated doxorubicin [23]. In this series, 141 elderly patients with DLBCL were compared by treatment regimen. Several important points in this study deserve mention regarding the patients' characteristics. There were two differences in the patients' characteristics that could have negatively influenced the RCOP group. First, patients in this group were older and had a worse performance status as follows: 55% were ECOG > 2 compared with 28.3% and 29.5% in the RCHOP and RChOP groups, respectively. Second, a higher proportion of patients with type 2 diabetes mellitus (*p* = 0.042) was observed in the RCOP group (32.5%), followed by the RChOP (25%) and RCHOP (11.32%) groups. The presence of other comorbidities, cardiovascular diseases in particular, was similar between these groups of patients; similarly, ejection fraction did not differ between the groups.

The proportion of patients with a poor prognosis, defined by the IPI score in this age group, ranged from 21% to 69% in multiple series [11, 16, 18]. We found that 60% of our patients had intermediate-high and high scores according to this prognostic scale; the survival for high-risk patients at 3 years is 59%, and we documented median OS of 34.7, 29.9, and 24.7 months in Groups A, B, and C, respectively.

Different authors have reported a better prognosis in the GC subtype of DLBCL. We documented more cases of germinal center subtypes in the RCHOP group, which may have influenced the better outcome of this group, although we should consider that this is a retrospective trial and that this variable was not considered when assigning treatment. Additionally, the higher frequency of GC subtype in this subgroup differs from the literature, as Mareschal et al. and Thunberg et al. [17, 20], using expression profiling techniques, demonstrated that elderly patients (≥80 years) are more common in the “nongerminal center subgroup” (non-GC) versus germinal center (GC) in younger patients (50–59 years) (*p* = 0.02).

The comparative effectiveness of anthracycline-containing chemotherapy has been previously assessed by different authors [11–14, 16, 24, 25], but there is little specific information regarding elderly patients, even less about the very elderly (80 years and older). Most studies, such as ours, were retrospective [11, 13, 16, 18, 23] and the majority of them included very few patients [13, 14] or are studies combining data from at least three or four centers. One of the largest studies is the one reported by Carson et al. [25], who analyzed 476 patients who received RCHOP; only 42% received an anthracycline-based regimen, 18% received treatment without an anthracycline, and 40% received no systemic treatment at all, with a median overall survival of 28.1 and 13.1 months for patients receiving treatment with and without anthracycline, respectively. Our results documented a higher mean OS in patients receiving full-dose RCHOP, and, in fact, in this group median OS was not achieved. In contrast, those who received reduced-dose anthracycline (Group B) and no anthracycline (Group C) had median OS of 22 and 19.5 months, respectively.  Table 5 compares our results to the response rates of retrospective series and prospective trials.

In terms of toxicity, the risk of myelosuppression and febrile neutropenia was decreased with the reduction of anthracycline [11]. In this study, the frequency of febrile neutropenia was 10% in patients treated without anthracycline, compared to 22% in both groups receiving doxorubicin. The frequency of transfusion requirements was similar between the three groups.

Although the ECOG scale may be useful for determining performance status in the general population, a geriatric assessment tool is recommended for elderly people. In the study of Spina et al., who classified patients into three different groups—fit, fragile, and unfit—the best responses were obtained from the fit and the fragile groups [26]. Most recent studies are conducted based on the main characteristics of the type of patients in an effort to have the most suitable therapeutic approach for each individual patient [12, 15, 24, 27, 28]. In this series, performance status had also a significant influence on OS.

Finally, this and most of the studies discussed in this paper are based on R-CHOP and CHOP-like regimens. We expect in the upcoming years that interventional phase 3 trials testing new molecules will focus on patients aged 70 to 80 years and older with the purpose of helping this growing population.

## Figures and Tables

**Table 1:** Comparison of demographic and clinical characteristics by treatment regimen.

	RCHOP	RChOP	RCOP	*p* value
*N* (%)	53 (100)	48 (100)	40 (100)	

Mean age (range)	68 (66–73)	75 (71.5–79.7)	77.5 (74–81)	<0.001^+^

Female : male	31 : 22	30 : 18	24 : 16	0.9^*∗*^

Presence of				
Cardiopathy	6 (11.32)	2 (4.17)	3 (7.5)	0.47^*∗*^
Mean FEVI ± SD	60.34 ± 5.87	61.27 ± 5.18	61.3 ± 5.67	0.649^*∗*^
Diabetes mellitus	6 (11.32)	12 (25)	13 (32.5)	0.042^*∗*^
Blood hypertension	14 (26.46)	13 (27.08)	14 (35)	0.62^*∗*^
Hepatitis (either HBV/HCV)	1 (1.89)	4 (8.33)	6 (15)	0.065^*∗*^
HIV	3 (5.66)	0	1 (2.5)	0.229^*∗*^
B symptoms	32 (60.38)	34 (70.83)	22 (55)	0.28^*∗*^
Bulky disease	25 (47.1)	27 (56.25)	16 (40)	0.31^*∗*^

ECOG	—	—	—	
1	38 (71.7)	34 (70.83)	18 (45)	0.05^*∗*^
2	11 (20.75)	11 (22.92)	15 (37.5)	
3	4 (7.55)	3 (6.25)	7 (17.5)	

Extranodal sites	—	—	—	
1	20 (37.74)	16 (33.3)	18 (45)	
2	10 (18.87)	8 (16.67)	6 (15)	0.95^*∗*^
>3	6 (11.32)	6 (12.49)	4 (10)	
None	17 (32.07)	18 (37.5)	12 (30)	

Ann Arbor stage	—	—	—	
I	3 (5.6)	6 (12.5)	5 (12.5)	
II	11 (20.75)	10 (20.83)	7 (17.5)	0.85^*∗*^
III	16 (30.19)	10 (20.83)	10 (25)	
IV	23 (43.4)	22 (45.83)	18 (45)	

Elevated LDH	25 (47.17)	27 (56.25)	18 (45)	0.519^*∗*^

Age adjusted IPI	—	—	—	
Low	10 (18.87)	9 (18.75)	5 (12.5)	
Low-intermediate	12 (22.64)	10 (20.83)	9 (22.5)	0.856^*∗*^
High-intermediate	14 (26.42)	17 (35.42)	11 (27.5)	
High	17 (32.08)	12 (25)	15 (37.5)	

Histological subtype	—	—	—	
Germinal center	29 (54.72)	14 (29.21)	16 (40)	0.02^*∗*^
Nongerminal center	17 (32.08)	26 (54.17)	17 (42.5)	
Unclassifiable	0 (0)	5 (10.37)	5 (12.5)	
Not available (not able to classify)	7 (13.2)	3 (6.25)	2 (15)	

^+^Kruskal-Wallis test.

^*∗*^Chi-square test.

**Table 2:** Complications, response rate, and survival by treatment regimen.

	RCHOP	RChOP	RCOP	*p* value
*N* (%)	53 (100)	48 (100)	40 (100)	—

Complications of treatment				

Infections	—	—	—	
None	18 (33.96)	14 (29.17)	23 (57.5)	**0.09**
Ambulatory treated	15 (22.64)	12 (25.0)	10 (25)	
Febrile neutropenia (FN)	12 (22.64)	11 (22.92)	4 (10)	
Other than FN, requiring hospitalization	8 (15.09)	11 (22.92)	3 (7.5)	

Myelosuppression	—	—	—	
None	36 (67.92)	30 (62.5)	26 (65)	0.94
No transfusion required	6 (11.32)	8 (16.67)	5 (12.5)	
Required transfusion	11 (20.75)	10 (20.83)	9 (22.5)	

Other complications (G III-IV)	—	—	—	
None	28 (52.83)	17 (35.42)	24 (60)	0.359
Pain	3 (5.66)	4 (8.33)	2 (5)	
2nd malignancy	0	1 (2.08)	0	
Neuropathy	1 (1.89)	5 (10.42)	2 (5)	
Lung	0	1 (2.08)	0	
Thrombosis	1 (1.89)	3 (6.25)	0	
Cardiovascular	8 (15.09)	5 (10.42)	6 (15)	
Gastrointestinal	8 (15.09)	5 (10.42)	6 (15)	
Renal	4 (7.55)	7 (14.58)	2 (5)	

Required radiotherapy	22 (41.51)	17 (35.42)	18 (45)	0.065

Response	—	—	—	
Complete	35 (66.04)	30 (62.5)	21 (52.5)	
Partial	6 (11.32)	3 (6.25)	3 (7.5)	0.418
Stable disease	0	0	1 (2.5)	
Progressive disease	2 (3.77)	6 (12.5)	3 (7.5)	
Not evaluated	10 (18.87)	9 (18.75)	12 (30)	

Relapsed	6 (11.32)	6 (12.5)	7 (17.5)	0.668

Mean disease-free survival (95% IC)	34.72 (29.54–39.89)	27.77 (22.28–33.25)	24.99 (18.9–31.09)	>0.05^*∗*^

Mean overall survival (95% IC)	35.8 (30.56–41.05)	27.69 (22.18–33.2)	44.29 (32.65–55.93)	>0.05^*∗*^

^*∗*^Restricted mean.

**Table 3:** Cox regression analysis of overall survival (OS).

Variable	HR	IC 95%	*p* value
Univariate analysis			

Treatment			
RCHOP	—		
RChOP	1.717	0.884–3.335	0.110
RCOP	1.844	0.928–3.664	0.081

ECOG			
1	—		
2	3.478	1.929–6.269	0.000
3	5.361	2.466–11.657	0.000

Infections			
None	—		
Ambulatory treated	0.519	0.199–1.351	0.179
Required hospitalization	3.402	1.659–6.977	0.001
Febrile neutropenia	3.646	1.808–7.352	0.000

B symptoms			
None	—		
Yes	2.267	1.213–4.236	0.010

Extranodal sites			
1	1.703	0.842–3.443	0.138
2	2.393	1.088–5.260	0.030
3 or more	2.039	0.802–5.182	0.134

Bulky disease			
Yes	1.948	1.125–3.372	0.017

Cycles of treatment	0.642	0.573–0.720	0.000

HB mg/dL			
>13			
10–12.9	1.431	0.777–2.636	0.249
8–10	2.543	0.774–8.355	0.124
<8	3.196	1.320–7.737	0.010

DHL			
High	2.012	1.157–3.496	0.013

Creatinine (mg/dL)	1.847	1.064–3.206	0.029

B2 microglobulin	1.196	1.061–1.350	0.003

Leucocytes	1.0001	1.00007–1.0002	0.000

Albumin (g/dL)	0.422	0.281–0.632	0.000

Age			
<75	—		
≥75	1.06	0.624–1.82	0.812

Multivariate analysis			

Treatment			
RCHOP	—		
RChOP	1.354	0.655–2.798	0.413
RCOP	1.045	0.425–2.568	0.922

ECOG			
1	—		
2	1.862	0.900–3.853	0.093
3	3.740	1.428–9.793	0.007

Infections			
None	—		
Ambulatory treated	0.557	0.198–1.561	0.266
Required hospitalization	1.971	0.759–5.112	0.163
Febrile neutropenia	2.550	1.029–6.317	0.043

B symptoms			
None	—		
Yes	2.377	1.150–4.912	0.019

Extranodal sites			
1	1.590	0.689–2.667	0.276
2	1.946	0.752–5.035	0.170
3 or more	3.637	1.269–10.422	0.016

Bulky disease			
Yes	1.895	0.895–4.011	0.095

Cycles of treatment	0.600	0.513–0.702	0.000

**Table 4:** Cox regression analysis of disease-free survival (DFS).

Variable	HR	IC 95%	*p* value
Univariate analysis			

Treatment			
RCHOP	—		
RChOP	1.380	0.442–4.302	0.578
RCOP	2.131	0.714–6.356	0.175

Infections			
None	—		
Ambulatory treated	0.761	0.315–1.838	0.545
Required hospitalization	3.152	1.457–6.819	0.004
Febrile neutropenia	4.481	2.222–9.035	0.000

Hb mg/dL			
>13	—		
10–12.9	1.555	0.854–2.831	0.148
8–10	1.669	0.398–6.999	0.484
<8	3.206	1.324–7.762	0.010

IPI			
Low	—		
Low-intermediate	1.721	0.598–4.954	0.314
High-intermediate	2.737	1.020–7.342	0.045
High	2.705	1.009–7.252	0.048

Cycles of treatment	0.661	0.589–0.740	0.000

B symptoms			
Yes	2.099	1.140–3.863	0.017

Albumin (g/dL)	0.427	0.285–0.641	0.000

ECOG			
2	2.328	1.282–4.227	0.005
3	3.900	1.767–8.605	0.001

LDH			
Increased	1.891	1.093–3.270	0.023

Creatinine (mg/dL)	1.965	1.130–3.415	0.017

Age	—	—	—
<75 years	—	—	—
≥75 years	1.2	0.703–2.048	0.503

B2M microglobulin	1.211	1.077–1.361	0.001

Leucocytes	1.0001	1.00005–1.0002	0.002

Multivariate analysis			

Treatment			
RCHOP	—		
RChOP	1.339	0.416–4.303	0.624
RCOP	11.967	2.781–51.483	0.001

Infections			
None	—		
Ambulatory treated	0.848	0.327–2.198	0.736
Required hospitalization	2.459	0.970–6.237	0.058
Febrile neutropenia	2.650	1.135–6.189	0.024

HB mg/dL			
>13			
10–12.9	2.008	1.053–3.827	0.034
8–10	2.121	0.468–9.606	0.329
<8	2.509	0.874–7.201	0.087

IPI			
Low	—		
Low-intermediate	3.397	1.011–11.414	0.048
High-intermediate	9.612	2.952–31.293	0.000
High	9.411	2.946–30.069	0.000

Cycles of treatment	0.558	0.470–0.6625	0.000

**Table 5:** Comparison of our series with other trials.

References	Age (M)/ *N*	IPI (HSP)	Treatment variations	Patients (%)	OR	CR	OS	PFS/EFS
Marchesi et al. 2013 (R) [16]	(78) 73	32.8%	TWCI	36 (49.3%)	91.2%	67.6%	55.5% at 2 yr	47.2% at 2 yr
			R-CHOP FD	22 (30.1%)	—	—	—	—
			R-CHOP AD	14 (19.1%)	—	—	—	—
			R-CVP CV	37 (50.7%)	69.7%	42.4%	24.3%	21.6%

Italiano et al. 2005 (R) [14]	(83) 29	12.5%	CHOP AD	22 (92%)	79%	62.5%	63% at 2 yr	50% at 2 yr

Yoshida et al. 2016 (R) [18]	(72) 135	69%	CHOP	46 (34%)	82.6%	76.1%	82.1% at 2 yr	72.5% at 2 yr
			THP-COP	69 (51%)	81.2%	63.8%	67.6% at 2 yr	64.8% at 2 yr
			Surgery	3 (2%)	—	—	—	—
			Palliative	17 (13%)	—	—	—	—

Nabhan et al. 2012 (R) [2]	(84) 170	62%	None	100%	72%	50%	44% at 4 yr	31% at 4 yr

Peyrade et al. 2011 (R) [11]	(83) 149	40%	R-CHOP AD	100%	73%	62%	59% at 2 yr	47% at 2 yr

Carson et al. 2015 (R) [25]	(83) 476	11%	AT	198 (41%)			28.1 mo	
			WA	87 (18%)			13.1 mo	
			NT	191 (40%)			1.9 mo	

Kreher et al. 2014 (R) [13]	(77) 30	27%	R-CHOP AD	100%	87%	60%	60% at 3 yr	49% at 3 yr

Spina et al. 2012 (P) [26]	(75) 100	5%	Fit (dose at 100%)			83%	54% at 5 yr	84% at 5 yr
			Fragile (75% reduction)			80%	61% at 5 yr	67% at 5 yr

Olivieri et al. 2012 (P) [23]	(74) 91	29.7%	R-CHOP 21	54 (59%)	—	81.5%	46% at 5 yr	—
			R-CHOP Doxo liposomal	22 (25%)	—	64%	31%	—
			R-mini-CHOP	15 (16%)	—	60%	41%	—

Coiffier et al. 2010 (P) [12]	(70) 202	15%	None	100%	82%	75%	58% at 5 yr	54% at 5 yr

Delarue et al. 2013 (P) [24]	(70) 602	13%	R-CHOP-21 (295 pts)	49%	86%	74%	67% at 3 yr	62% at 3 yr
		22%	R-CHOP 14 (304 pts)	51%	—	—	72%	60%

This series *N* = 141	68		R-CHOP	53	77.3%	66%	74% at 3 yr	70% at 3 yr
	75		R-ChOP	48	68.7%	62.5%	60%	54%
	77.5		R-COP	40	60%	52.5%	60%	52%

RTX: rituximab, Doxo: doxorubicin, CTX: cyclophosphamide, VCR: Vincristine, PDN: prednisone, AT: treatment with anthracyclines, WA: without anthracyclines, NT: no treatment, FD: full doses, AD: attenuated, CV: conservative, RI: reduced intensity, HSP: high score patients, and TWCI: treatment with curative intention, (R) retrospective and (P) prospective.

## References

[1] Ferlay J., Soerjomataram I., Ervik M. (2013). *GLOBOCAN 2012 v1.0, Cancer Incidence and Mortality Worldwide*.

[2] Nabhan C., Smith S. M., Helenowski I. (2012). Analysis of very elderly (≥80 years) non-hodgkin lymphoma: impact of functional status and co-morbidities on outcome. *British Journal of Haematology*.

[3] Varga C., Holcroft C., Kezouh A. (2014). Comparison of outcomes among patients aged 80 and over and younger patients with diffuse large B-cell lymphoma: A Population Based Study. *Leukemia and Lymphoma*.

[4] Cultrera J. L., Dalia S. M. (2012). Diffuse large B-cell lymphoma: current strategies and future directions. *Cancer Control*.

[5] Smith A., Crouch S., Lax S. (2015). Lymphoma incidence, survival and prevalence 2004–2014: Sub-type analyses from the UK's Haematological Malignancy Research Network. *British Journal of Cancer*.

[6] Fisher R. I., Gaynor E. R., Dahlberg S. (1993). Comparison of a standard regimen (CHOP) with three intensive chemotherapy regimens for advanced non-Hodgkin's lymphoma. *The New England Journal of Medicine*.

[7] Roschewski M., Staudt L. M., Wilson W. H. (2014). Diffuse large B-cell lymphoma—treatment approaches in the molecular era. *Nature Reviews Clinical Oncology*.

[8] Feugier P., Van Hoof A., Sebban C. (2005). Long-term results of the R-CHOP study in the treatment of elderly patients with diffuse large B-cell lymphoma: a study by the Groupe d'Etude des Lymphomes de l'Adulte. *Journal of Clinical Oncology*.

[9] Xue Q.-L. (2011). The frailty syndrome: definition and natural history. *Clinics in Geriatric Medicine*.

[10] Zelenetz A. D., Gordon L. I., Wierda W. G., Abramson J. S., Advani R. H., Andreadis C. B. (2014). Non-Hodgkin's lymphomas. Version 4. *Journal of the National Comprehensive Cancer Network*.

[11] Peyrade F., Jardin F., Thieblemont C. (2011). Attenuated immunochemotherapy regimen (R-miniCHOP) in elderly patients older than 80 years with diffuse large B-cell lymphoma: a multicentre, single-arm, phase 2 trial. *The Lancet Oncology*.

[12] Coiffier B., Thieblemont C., Van Den Neste E. (2010). Long-term outcome of patients in the LNH-98.5 trial, the first randomized study comparing rituximab-CHOP to standard CHOP chemotherapy in DLBCL patients: a study by the Groupe d'Etudes des Lymphomes de l'Adulte. *Blood*.

[13] Kreher S., Lammer F., Augustin D., Pezzutto A., Baldus C. D. (2014). R-split-CHOP chemotherapy for elderly patients with diffuse large B-cell lymphoma. *European Journal of Haematology*.

[14] Italiano A., Jardin F., Peyrade F., Saudes L., Tilly H., Thyss A. (2005). Adapted CHOP plus rituximab in non-Hodgkin's lymphoma in patients over 80 years old. *Haematologica*.

[15] Peyrade F., Gastaud L., Ré D., Pacquelet-Cheli S., Thyss A. (2012). Treatment decisions for elderly patients with haematological malignancies: a dilemma. *The Lancet Oncology*.

[16] Marchesi F., Cenfra N., Altomare L. (2013). A retrospective study on 73 elderly patients (≥75 years) with aggressive B-cell non Hodgkin lymphoma: clinical significance of treatment intensity and comprehensive geriatric assessment. *Journal of Geriatric Oncology*.

[17] Mareschal S., Lanic H., Ruminy P., Bastard C., Tilly H., Jardin F. (2011). The proportion of activated B-cell like subtype among de novo diffuse large B-cell lymphoma increases with age. *Haematologica*.

[18] Yoshida M., Nakao T., Horiuchi M. (2016). Analysis of elderly patients with diffuse large B-cell lymphoma: aggressive therapy is a reasonable approach for ‘unfit’ patients classified by comprehensive geriatric assessment. *European Journal of Haematology*.

[19] Common Terminology Criteria for Adverse Events (CTCAE) http://ctep.cancer.gov/protocolDevelopment/electronic_applications/ctc.htm.

[20] Thunberg U., Enblad G., Berglund M. (2012). Classification of diffuse large B-cell lymphoma by immunohistochemistry demonstrates that elderly patients are more common in the non-GC subgroup and younger patients in the GC subgroup. *Haematologica*.

[21] Meignan M., Gallamini A., Haioun C. (2015). Report on the 5th International Workshop on Positron Emission Tomography in Lymphoma held in Menton, France, 19-20 September 2014. *Leukemia & Lymphoma*.

[22] Cheson B. D., Pfistner B., Juweid M. E. (2007). Revised response criteria for malignant lymphoma. *Journal of Clinical Oncology*.

[23] Olivieri A., Gini G., Bocci C. (2012). Tailored therapy in an unselected population of 91 elderly patients with DLBCL prospectively evaluated using a simplified CGA. *Oncologist*.

[24] Delarue R., Tilly H., Mounier N. (2013). Dose-dense rituximab-CHOP compared with standard rituximab-CHOP in elderly patients with diffuse large B-cell lymphoma (the LNH03-6B study): a randomised phase 3 trial. *The Lancet Oncology*.

[25] Carson K. R., Riedell P., Lynch R. (2015). Comparative effectiveness of anthracycline-containing chemotherapy in United States veterans age 80 and older with diffuse large B-cell lymphoma. *Journal of Geriatric Oncology*.

[26] Spina M., Balzarotti M., Uziel L. (2012). Modulated chemotherapy according to modified comprehensive geriatric assessment in 100 consecutive elderly patients with diffuse large B-cell Lymphoma. *Oncologist*.

[27] Gimeno E., Sánchez-González B., Álvarez-Larrán A. (2011). Intermediate dose of nonpegylated liposomal doxorubicin combination (R-CMyOP) as first line chemotherapy for frail elderly patients with aggressive lymphoma. *Leukemia Research*.

[28] Habermann T. M., Weller E. A., Morrison V. A. (2006). Rituximab-CHOP versus CHOP alone or with maintenance rituximab in older patients with diffuse large B-cell lymphoma. *Journal of Clinical Oncology*.

